# The Homœopathic Convention of 1881

**Published:** 1881-10

**Authors:** P. P. Wells

**Affiliations:** Brooklyn, N. Y.


### HOMŒOPATHIC PHYSICIAN.

A MONTHLY JOURNAL OF MEDICAL SCIENCE.

"If our school ever gives up the strict inductive method of Hahnemann, we are lost, and deserve to be mentioned only as a caricature, in the history of medicine."—CONSTANTINE HERING.

Vol. I. OCTOBER, 1881. No. 10.

### THE INTERNATIONAL HOMŒOPATHIC CONVENTION OF 1881.

P. P. WELLS, M. D., BROOKLYN, N. Y.

IN 1876, the writer of this paper was told that the above-named body would assemble in London, in July of this present, or of the last year, and would be made up of the leading minds of the homœopathic school, from this and the many countries in Europe where homœopathy is studied and practiced, so far as it might be practicable to gather them at the appointed time. The prospective view of these, so gathered, was a pleasant vision, and so it continued to be till it met, acted and adjourned, in July of the present year. It was pleasant because such a gathering of minds from so many countries, who had received the God-given law of healing as that which in its philosophy and practice contains all the world posesses of a "science of therapeutics," it was supposed would be earnestly and wholly employed in raising this law and its philosophy into a clearer light, and in giving to the world a broader and brighter vision of its truth, its promise of blessing to the sick, and an increased knowledge of its past grand achievements in practical healing. In short, it was pleasant to believe that as the fruit of this gathering, homœopathy would be raised to a higher plane, as a science, and there be placed in so clear a light that all the world might the better understand that this which we so greatly love, trust and practice is indeed God-given, and not the outgrowth from any human imagination, however brilliant; that it is indeed concrete truth in itself, and not existing merely in the acceptance or opinion of any man or class of men. It certainly was not unreasonable to expect this from such a body as this Convention was to be. The world, and notably that part of it which is represented by the practitioners of our school not present in this Convention, had a right to expect and demand this at the hands of those who were. The object of this paper will be to see, from the report of their doings, how far this reasonable expectation has been met. What, if anything, has it added to our knowledge of our law or its philosophy? what has it given to the absent world by its meeting and labors calculated to strengthen its confidence in the truth, value or efficacy of the law? for it was innocently supposed by many of these absentees that to do something of this kind was the especial object of the Convention. Indeed, the President declared the first object of these meetings to be "the consideration of the best plans for propagating the *method* of Hahnemann." That homœopathy was "a *method* and not a doctrine or system." Thus he began the work of degrading that which has been long recognized as a *law* existing in the nature of man, there planted by his Creator, in which exists the relationship between drug agencies and sicknesses, by which the one was made the curative of the other, to the low position of a "*method.*"

The passage of a certain resolution at Albany, in 1878, by the New York State Homœopathic Medical Society, degrading this law to the level of a mere *rule* of practice, like any other rule which any man might set up for himself with benevolent intent, was characterized at the time by the writer as a crime against science. In his judgment of this proceeding, he is now, as then, fully convinced of the justice of this sentence. It was then objected to by the excellent and genial friend who has just presided at this last Convention, because, he said, "this is just what I have been doing all my life." I have no doubt this confession is true, and the more so because of this his last effort to reduce our *law* to a mere "*method*" of practice. We did not reply to Dr. Hughes' objection to our view of the criminal character of the Albany proceeding, in the first place, because we did not see how his objection changed in any degree the character of the Albany transaction. If criminal before, it was after his objection and the interposition in its favor of his own life-long example. We now reply to his objection to our judgment, and to this, his last act, in accord with that which he said he had "been doing all his life," that neither his personal example, excellent as we have no doubt this has even been in other matters, nor repetitions of acts, however often these may have been, has any power to sanctify crime—crime remains crime after a thousand repetitions, the last the same as the first.

The second object of the Convention, as given by the President, was "the development of homœopathy." If this were to be gained by in the first place reducing its law to a mere method, it is submitted that development so initiated, would be in a direction downward and backward, and from the grandly magnificent and universal in its application, to the little and unimportant, and not likely long to be of interest to any one, because of its utter insignificance.

By way of "developing homœopathy,"—the second object of the Convention—and belittling its author, which were, apparently, too nearly identical in the minds of some prominent members to be pleasant to the contemplation of those who believe both in homœopathy and its author, the President informs the Convention that there was, in Hahnemann's time, "no homœopathic physiology." Was this given to his friends present as an important piece of information it were well they should know? Or was it to express to them the President's view of the poverty of the time of the birth of our system of philosophy and practice (for such we maintain our law and its philosophy is), in professional scientific possessions? In either case, it would be pleasant to know just what "*homœopathic* physiology" would be like, if the President's idea of it could be realized. We have been accustomed to consider homœopathy as the science of therapeutics, and therapeutics as having to do with sick life, and no other. Now, physiology is, as we understand it, the science of life in health, and is thus distinguished from pathology (of which some of our friends are just now so fond), which is the science of life sick. No "homœopathic physiology," indeed! Well, I do not know that they had a homœopathic trigonometry. But what then? The supposition of such a thing, and a need of it is no more absurd than to talk of homœopathic physiology.

And then, further, by way of "development," there was then "no such thing as homœopathic pathology." We take issue with the President on this statement, if by it he means that homœopathy, then as now, dealt or deals with the sick regardless of the patient's sick life. It is with this it is exclusively concerned in all its practical duties. It is this sick life which *is* pathology, and a knowledge of this is its science, and besides this there is no science of pathology in any time, other than in the imagination of self-deceived men. The author of homœopathy investigated the elements of sick life as man had never done before him, and those who to-day are his true followers and representatives interrogate these as no other men do, with a thoroughness unknown to those who are partially his followers, and which is entirely foreign to all practitioners of medicine who repudiate Hahnemann and his law, and prate and chatter, pathology! The necessity was on him, and is on his true followers, to carry his investigations of morbid phenomena to their utmost depths and to their very source, that the true nature of the affection to be treated may be known, and this because it can be known in no other way. It is only by a study of these elements that any idea of rational pathology can be conceived of. All outside of this, which is called pathology, has its existence solely in a deceived imagination. These elements, commonly known as symptoms, are the only medium of approach to any knowledge of a true pathology of any case. And yet it is too common for those who are loudest and most frequent in their talk of pathology to affect to deride these only guides to its true knowledge, or to a practice founded on law. It is by means of these despised symptoms that the true pathologist penetrates the deepest secrets of his case, and it is by these alone they can be reached. And yet we have heard, and not long since, from a presiding officer of another learned body, this practice, so guided to the depths and secrets of morbid conditions characterized as "superficial." He did not know that this, and this only, was the way to the profound. He called it "prescribing on the surface," when thus guided by these only guides. He, more than others, prescribes on the surface, who regardless of these true guides, generalizes a few phenomena and from these predicates a general condition, gives this a learned name, calls it the pathology of his case, and proceeds, if a partial homœopathist, to give a remedy he supposes to have produced a similar condition in its proving, and this double hypothesis and wholesale guessing he calls scientific homœopathic prescribing. He is the man on the surface, and even his most vigorous guessing is not likely to carry him often much below it. The true follower of the master thus pursues these elements of his case into its intimate pathology, through the medium of its symptoms, because this can be reached in no other way and by no other means. And, further, because the like which cures, which to him is the one all important end of all his investigations, is only found in these elements which having disclosed to him its true pathology, now further show him his curative in the drug which has been found to produce on the healthy organism phenomena most like those of the case before him. "On the surface," indeed! Did the man know what he was talking about? No pathology in true homœopathy! It can hardly be too much to suggest that now we have heard from the feeble and the flippant more than enough of the lack of pathology in true homœopathy. If even these will give themselves the trouble to look for this where alone it can be found, they will find bright and solid facts, which they may profitably place in the stead of that they have vaunted, which consists so largely of hypothesis and imagination.

But, says the pseudo pathologist, this prescribing, under the guidance of symptoms only, reduces the physician to a mere *symptom coverer*. We reply, that this practice, perfected, exalts him to the highest position in the ranks of healers. This charge has been cast as a sneer even at such heroes in the practice and literature of our profession as Bœnninghausen and Hering, by those who were wholly incapable of either comprehending or imitating their method. A sneer was the highest reach of their capacity. They are not sufficiently intelligent to know that the exact covering of the symptoms of a case is the ultimate perfection of prescribing, under the law which is the sure foundation of the science of therapeutics. He is and has ever been the most successful healer who has been able to do this most perfectly.* Surely this man has no occasion to quail before this flippancy of ignorant or prejudiced incompetents. The man who can do this, can stand by the side of that peerless prescriber of Münster, and challenge the world to produce another, of whatever other school of medicine, with a record of equal success in dealing with the gravest forms of human maladies. Symptom coverer, indeed! The most exalted characters have been a mark for the spite of the weak and the wicked. And thus these most exalted prescribers we have named, have not escaped. The sneer which was intended as an insult, was really the brightest jewel in their crown.

In a paper read by Dr. Hughes, entitled "Generalization and Individualization," he further attempted the development of homœopathy, by giving to the former the first and almost exclusive importance in prescribing, except for a class of affections, notably those

*It is admitted that this manner of prescribing, while it is the beau ideal of clinical practice, is at the same time one of the most difficult of human duties. Indeed, there have been few who could fully realize this perfectly in their practice. Presumably these sneerers are not included in this comparatively small number.

of a nervous character, which even the ingenuity of our modern pseudo pathologists, have not been quite equal to moulding into forms sufficiently definite to enable them to generalize these as they would like, and as they seem to think they have, the rest of human maladies. He brings Hahnemann as authority for the generalization he advocates, in that he recognized certain forms of disease which recur with such uniformity of elements as to establish a relationship of curative to them of a given drug, whenever these diseases may appear. It cannot be denied that the doctor had a seeming of truth on his side in this presentation of the authority of the master for his heresy, for such we cannot but regard his advocacy of generalization to the exclusion of the individualization which Hahnemann everywhere advocated and practiced. We say he has a seeming of truth, for he has only this. It was by this very individualization which the doctor would reduce to a second rank in importance in finding a simillimum, that the master reached his conclusions as to his supposed relationship of copper to cholera, for example, and so of the other instances given in the paper. It was only by the strictest analysis and individualization of the elements of the diseased and drug action that he discovered the relationship, in the light and under the guidance of the law of similars. With him it was, with the epidemic of cholera, for example, as it was with every individual case of disease, the simillimum was sought and found by analysis and comparison, and, in no other way, each case and each symptom being carefully studied as an individuality. Indeed, if we take from homœopathy this *sine qua non*, we take from it the very life and soul of this science of therapeutics, and it will be only a dead corpse, and needing but a coffin and a grave to be buried out of sight, to be speedily forgotten, as so worthless a thing would well deserve to be. Deprived of this, and homœopathy is deprived of its identity. The attempt to develop its science by degrading this, its one most essential feature, is to attempt to run it into the ground and into forgetfulness in the briefest possible time. Its friends may well pray for salvation from all such development.*

---

*The *Homœopathic World* says: "This paper was well handled by Dr. Drysdale, who pointed out that generalization stood for pathology. To this Dr. Hughes was understood to assent." Now, then, we know what it is, this of which "there was no such thing" in Hahnemann's time. It is generalization. It is well to know this at last, for hitherto this so much talked of and vaunted science has been a very indefinite and shadowy thing. It is generalization. Dr. Hughes admits this. But there was no pathology, *i. e.*, no generalization, in Hahnemann's day. The president and others said so. And yet the president says Hahnemann generalized, and if we may accept his statements, he seems at times to have done this to some pur-

If we look into papers read in the Convention by other gentlemen, we find in each, or nearly so, something to approve and often much to condemn. In many there is an attempt to improve that in our philosophy, practice or methods which needs no mending. In this there appears at times a disposition to be meddling, and almost always in a way at the expense of Hahnemann's reputation or that of his system of practical medicine, these gentlemen were supposed to have accepted and approved, and to the development of which we were assured, and certainly we hoped, the labors of this Convention were to be devoted. We are compelled to say the issue in no way or degree justifies the promise. There were at times, in some of the papers read, an apparent inkling of homœopathic philosophy, but, lest this should shine with too great brightness, the writer immediately discovers that its author was a dreamer, a fanatic, or an ignoramus, or at least a something very much below the station in science the writer was viewing himself as occupying, being at all times very careful to have it understood that he was not in any way to be regarded as unreasonably entangled with Hahnemann, or his dreamings, or with aught belonging to him. In this, it must be admitted they were, for the most part, quite successful. If at any time they were seeming to accept aught from the master it was oftener than otherwise to show how deftly they could mend it all, and perchance to hint how much better it might have been could the sage of Köthen have had the benefit of their counsel. The most startling among the many remarkable statements of papers read in the Convention was one by gentlemen from Belgium, making Hahnemann and Hering, among others, indorsers of alternation. It was never my fortune to know Hahnemann personally, therefore I can only say that his teachings, so far as I am acquainted with them, seem to me wholly at variance with this heresy which these gentlemen represent him as approving. With Hering it was different. I knew him well, and it was my great good fortune to possess his confidence. The last time I saw him alive he gave me his estimate of this heresy. If I could give to these gentlemen the emphasis and expression of disgust with which he said, "*that abomination*, alternation," I am sure they would not soon again represent him as its approver.

Some one has characterized the doings of this Convention as

---

pose. The President's logic seems a little loose. Has he been, in this generalization paper, weaving a fabric of his own, to which he has endeavored to attach Hanemann's trade-mark? However this may be, and whatever may be the product of the president's ingenuity, certainly homœopathy it is not.

largely devoted to "beating empty straw," which is well. They were given, to a considerable extent, to questions upon which all was said long ago, which could be profitably said, and we have been content to leave those who were not convinced by the arguments used for the right side, to go on enjoying their opinions of the wrong, to their heart's content. To talk reason to them was only to "beat empty straw," and this is not a dignified employment.

The Convention resolved it had had a pleasant time, and adjourned. In our gladness for their success in this particular, we cannot overcome our sense of their failure to accomplish anything our expectations looked for as the result of their gathering and labors.

